# Supplementary material for: A complex hydride lithium superionic conductor for high-energy-density all-solid-state lithium metal batteries
Source: Nat Commun. 2019 Mar 6;10:1081. doi: 10.1038/s41467-019-09061-9 (PMC6403359; doi:10.1038/s41467-019-09061-9)
Supplement: Supplementary file 1 — Supplementary Information [file 41467_2019_9061_MOESM1_ESM.pdf]

A complex hydride lithium superionic conductor for high−energy−density all−solid−state lithium metal batteries

Kim *et al*.

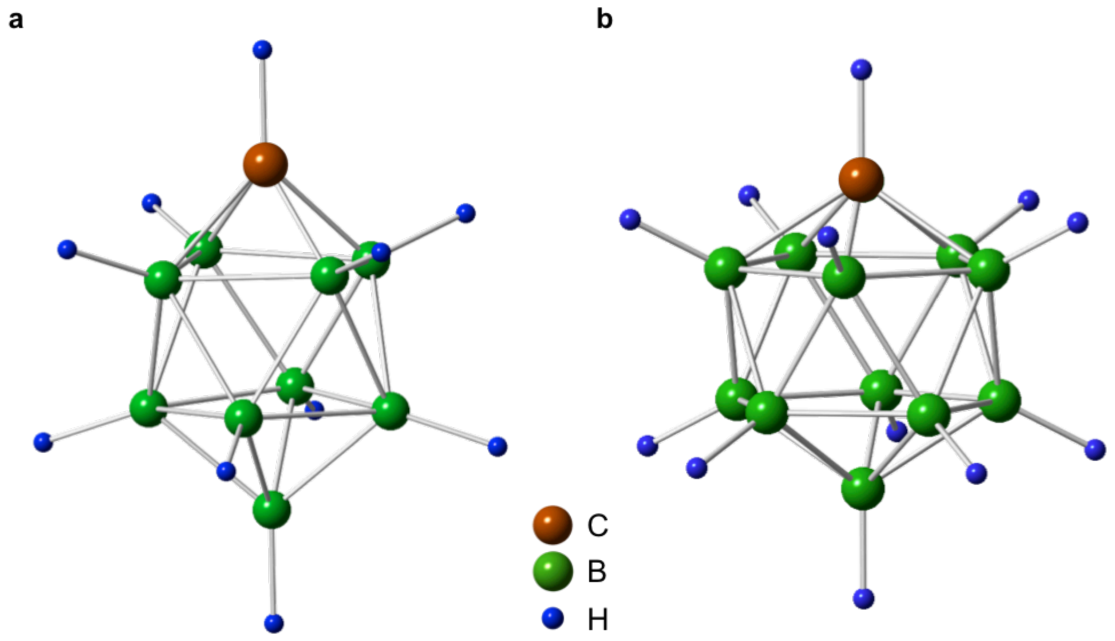

Supplementary Figure 1 | Structures of *closo*−type (cage−like) complex anions. a,b, Geometries of **(a)** $(CB_9H_{10})^-$ and **(b)** $(CB_{11}H_{12})^-$ complex anions. The brown, green, and blue spheres represent C, B, and H atoms, respectively.

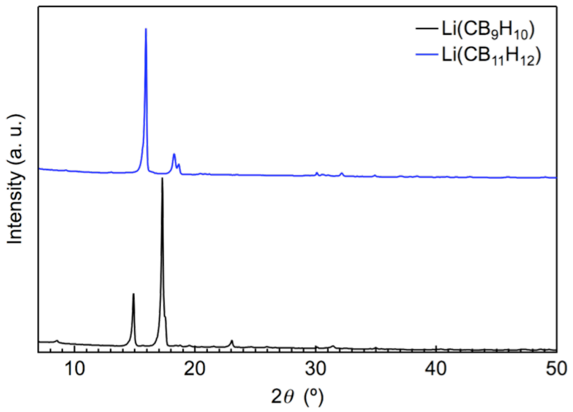

Supplementary Figure 2 | Structures of starting materials. XRD patterns of $Li(CB_9H_{10})$ and $Li(CB_{11}H_{12})$ at room temperature. Crystal systems, space groups, and lattice parameters are summarized in Supplementary Table 2.

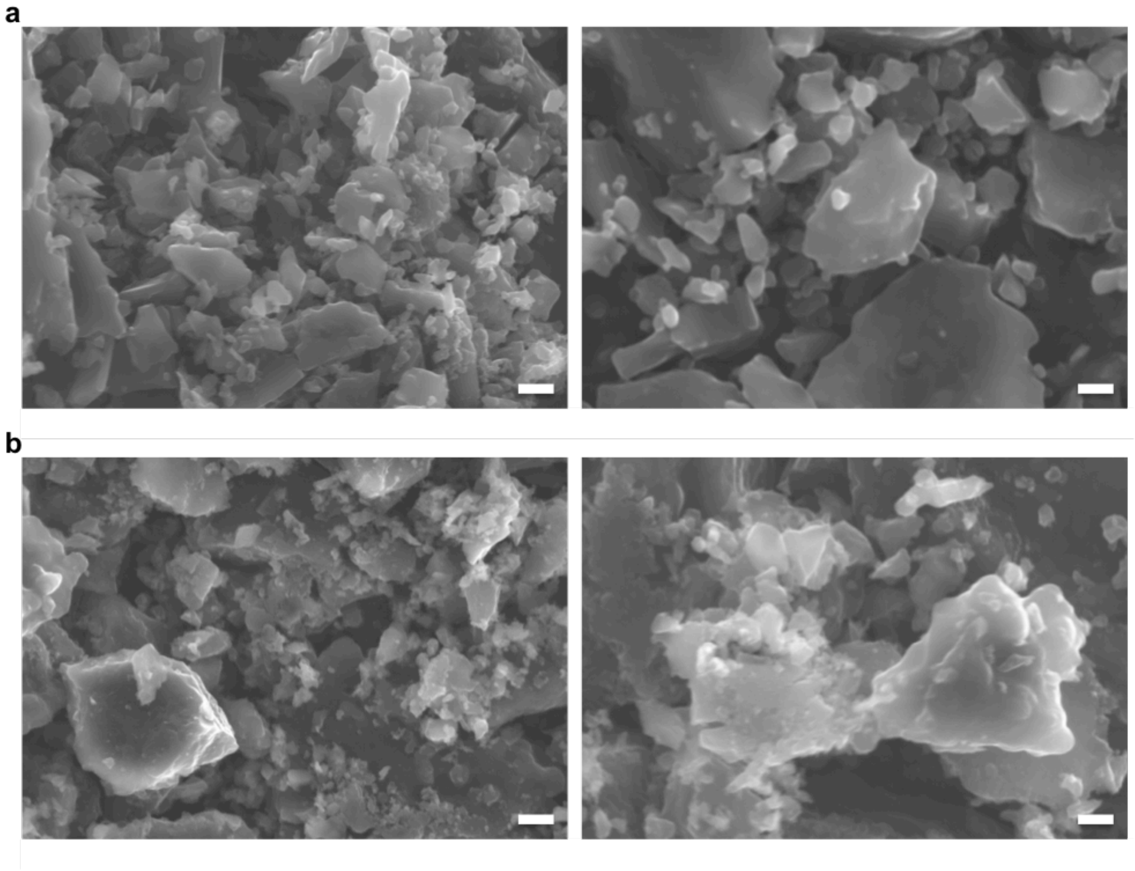

Supplementary Figure 3 | Morphologies and particle sizes of starting materials. a,b, SEM images of **(a)** $Li(CB_9H_{10})$ and **(b)** $Li(CB_{11}H_{12})$. Scale bars, 20 μm.

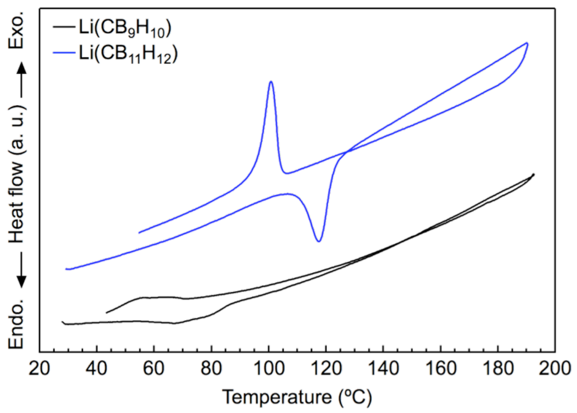

Supplementary Figure 4 | Phase transitions of starting materials. DTA profiles of $Li(CB_9H_{10})$ and $Li(CB_{11}H_{12})$. The phase transition temperatures are summarized in Supplementary Table 1.

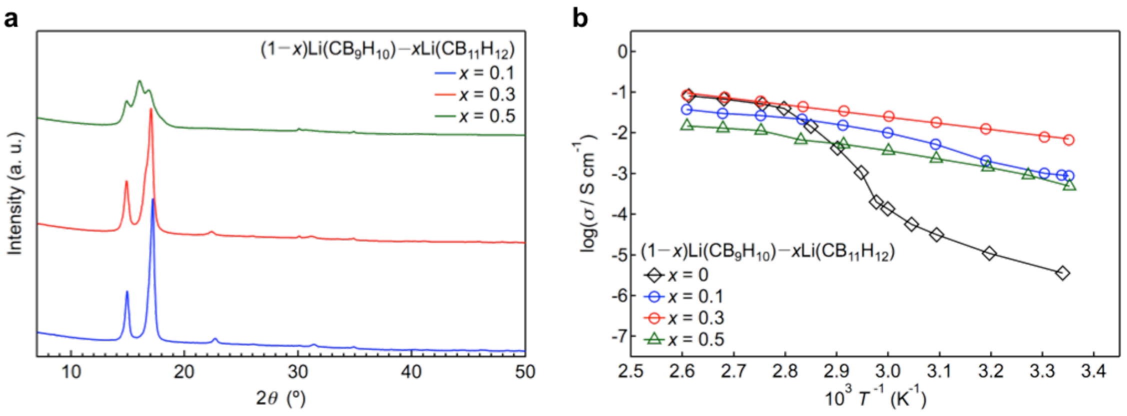

Supplementary Figure 5 | Structures and ionic conductivities of *x*Li(CB_9H_{10})−(1−*x*)Li($CB_{11}H_{12}$). a,b, (a) XRD profiles and **(b)** ionic conductivities of *x*Li(CB_9H_{10})−(1−*x*)Li($CB_{11}H_{12}$) (x = 0.1, 0.3, and 0.5).

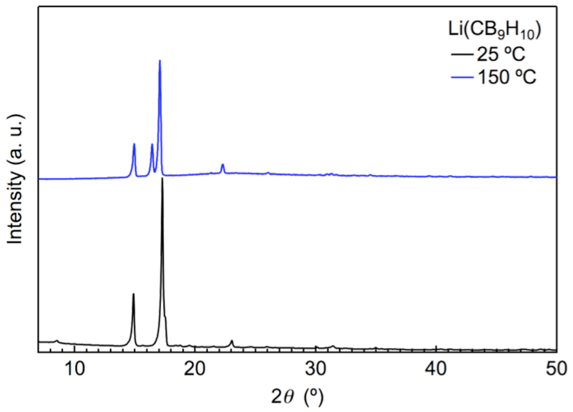

Supplementary Figure 6 | Structure change after phase transition of Li(CB_9H_{10}). XRD profiles of Li(CB_9H_{10}) at 25 and 150 °C. The high−*T* phase of Li(CB_9H_{10}) belongs to the space group *P*31*c* in the hexagonal symmetry[1]. Crystal systems, space groups, and lattice parameters are summarized in Supplementary Table 2.

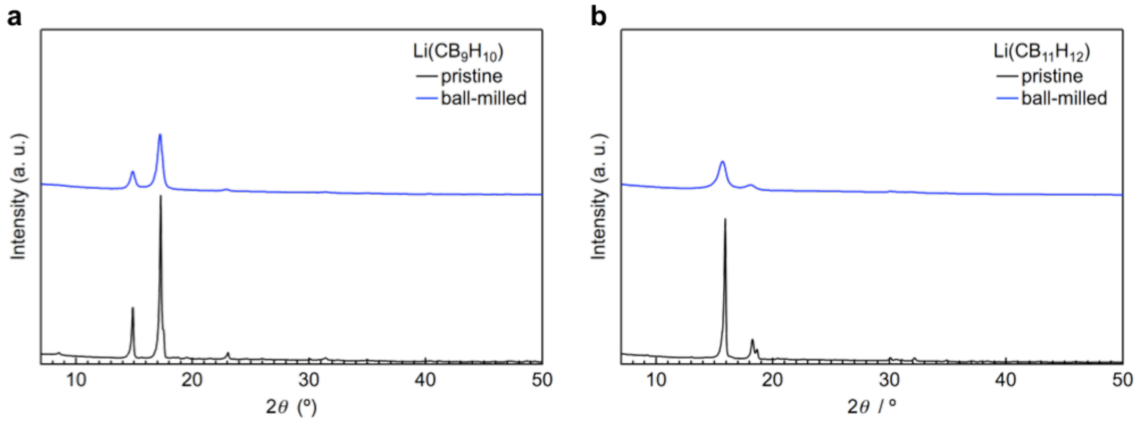

Supplementary Figure 7 | Structure change of starting materials after mechanical ball−milling. a,b, XRD profiles of **(a)** Li(CB_9H_{10}) and **(b)** Li($CB_{11}H_{12}$) before and after mechanical ball−milling at 400 rpm for 20 h.

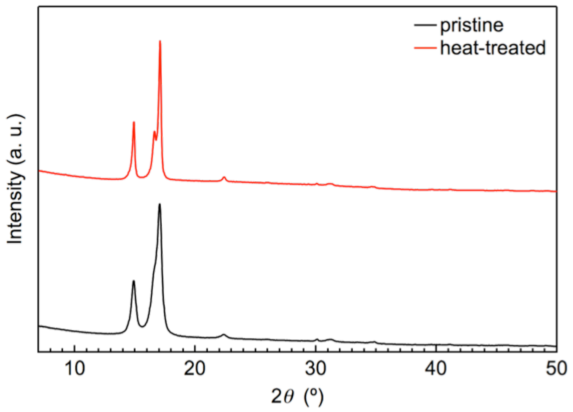

Supplementary Figure 8 | Thermal stability of 0.7Li(CB_9H_{10})–0.3Li($CB_{11}H_{12}$). XRD profiles of 0.7Li(CB_9H_{10})–0.3Li($CB_{11}H_{12}$) before and after heat–treatment at 473 K for 12 h.

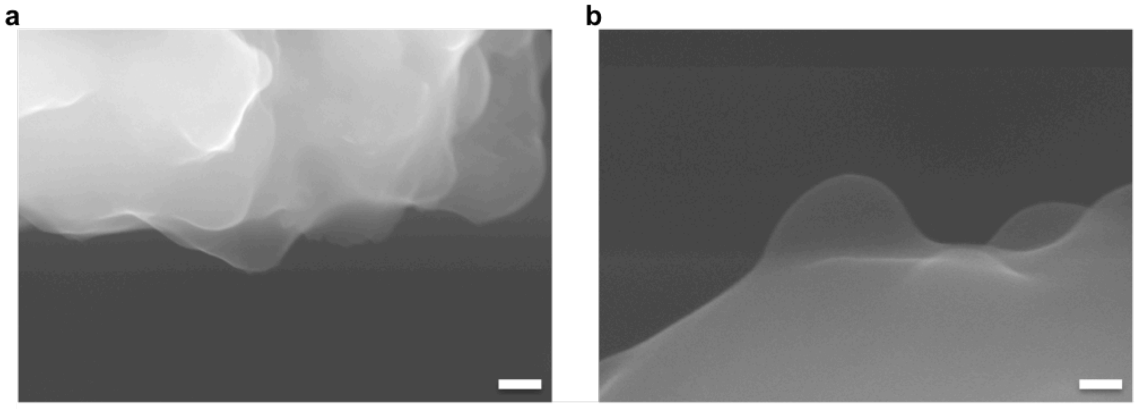

Supplementary Figure 9 | Morphology of the prepared sample. a,b, FE−SEM images of 0.7Li(CB_9H_{10})−0.3Li($CB_{11}H_{12}$). The 0.7Li(CB_9H_{10})−0.3Li($CB_{11}H_{12}$) particles are interconnected with very smooth edges. Scale bars, 1 μm.

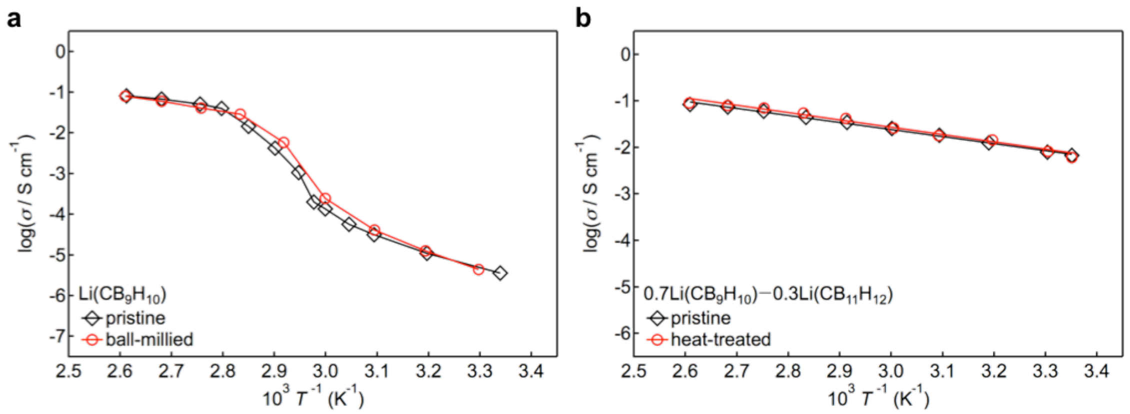

Supplementary Figure 10 | Effects of ball−milling and heat−treatment on conductivity. a,b, Ionic conductivities of **(a)** $Li(CB_9H_{10})$ before and after ball−milling at 400 rpm for 20 h and **(b)** $0.7Li(CB_9H_{10})-0.3Li(CB_{11}H_{12})$ before and after heat−treatment at 473 K for 12 h.

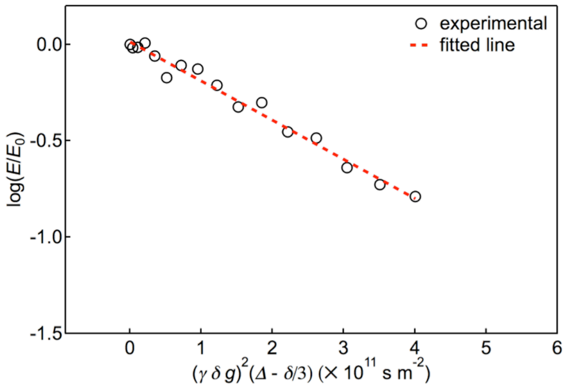

Supplementary Figure 11 | Lithium self–diffusion coefficient of 0.7Li(CB_9H_{10})–0.3Li($CB_{11}H_{12}$). Stimulated echo attenuation plot of 0.7Li(CB_9H_{10})–0.3Li($CB_{11}H_{12}$) at 25°C. The self–diffusion coefficient was estimated using the Stejskal and Tanner equation[2,3]. The diffusion time (Δ) was set to be short enough (20 ms) to avoid finite crystallite size effects. The pulsed field gradient time (δ) was fixed at 5 ms and the gradient strength (g) was varied from 36.2 to 900 G cm^{-1} (16 steps). ^{1}H NMR analyses confirmed no contribution of hydrogen diffusion to ionic diffusion.

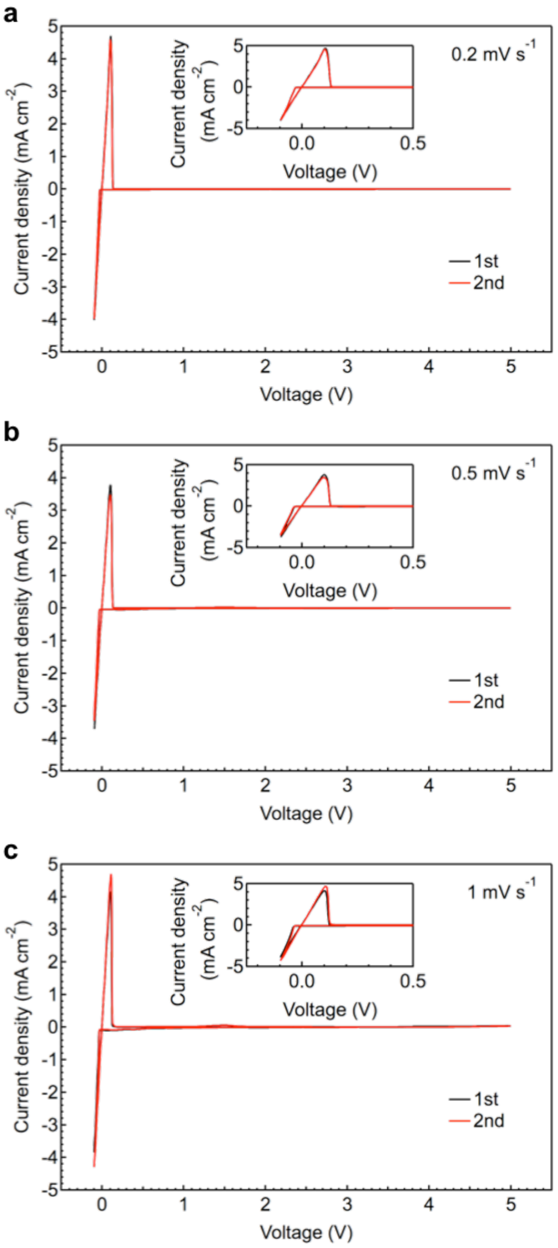

Supplementary Figure 12 | Electrochemical stability of 0.7Li(CB_9H_{10})−0.3Li($CB_{11}H_{12}$). a−c, CV curves of Mo/0.7Li(CB_9H_{10})−0.3Li($CB_{11}H_{12}$)/Li cells at scan rates of **(a)** 0.2 mV s^{-1}, **(b)** 0.5 mV s^{-1}, and **(c)** 1 mV s^{-1} during 2 cycles. Insets show the magnified plots in the low−voltage region.

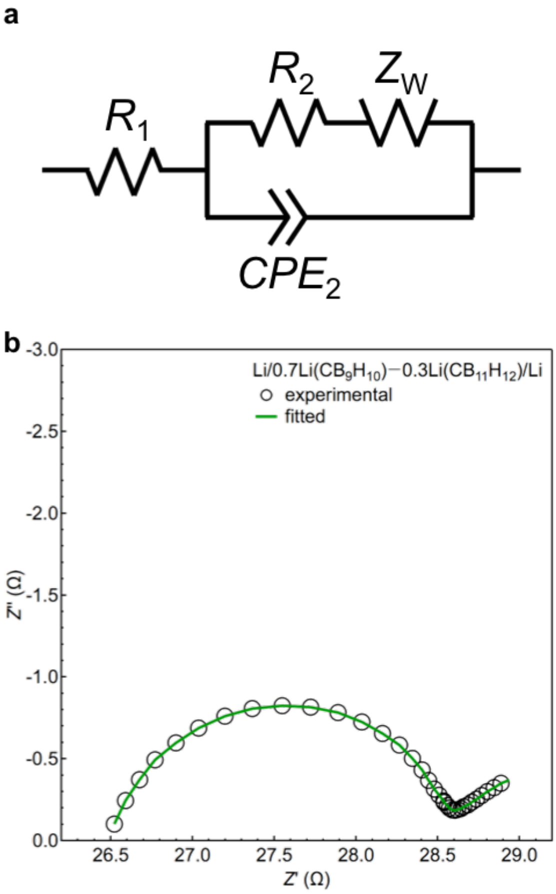

Supplementary Figure 13 | Interfacial resistance between the 0.7Li(CB_9H_{10})−0.3Li($CB_{11}H_{12}$) solid electrolyte and the lithium metal anode. a, Equivalent circuit used for fitting. **b,** Nyquist plot and fitted curve of a Li/0.7Li(CB_9H_{10})−0.3Li($CB_{11}H_{12}$)/Li cell.

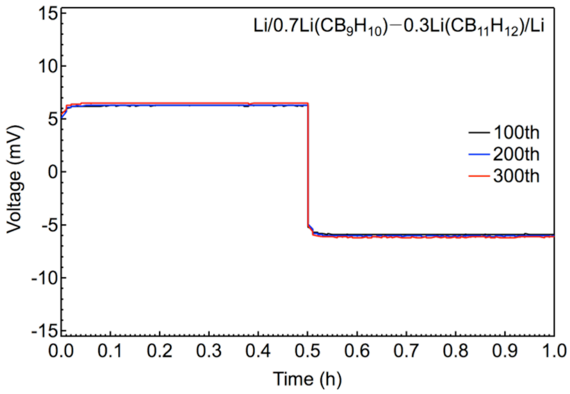

Supplementary Figure 14 | Stability of lithium ion transfer across the interface between the 0.7Li(CB_9H_{10})−0.3Li($CB_{11}H_{12}$) solid electrolyte and the lithium metal anode. Galvanostatic cycling profiles of a Li/0.7Li(CB_9H_{10})−0.3Li($CB_{11}H_{12}$)/Li cell at 0.2 mA cm^{-2}.

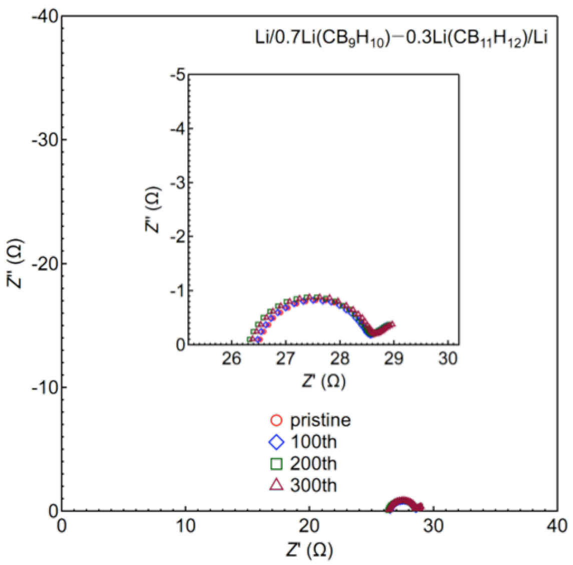

Supplementary Figure 15 | Interfacial resistances between the 0.7Li(CB_9H_{10})−0.3Li($CB_{11}H_{12}$) solid electrolyte and the lithium metal anode. Nyquist plots of a Li/0.7Li(CB_9H_{10})−0.3Li($CB_{11}H_{12}$)/Li cell before cycling and after 100, 200, and 300 cycles.

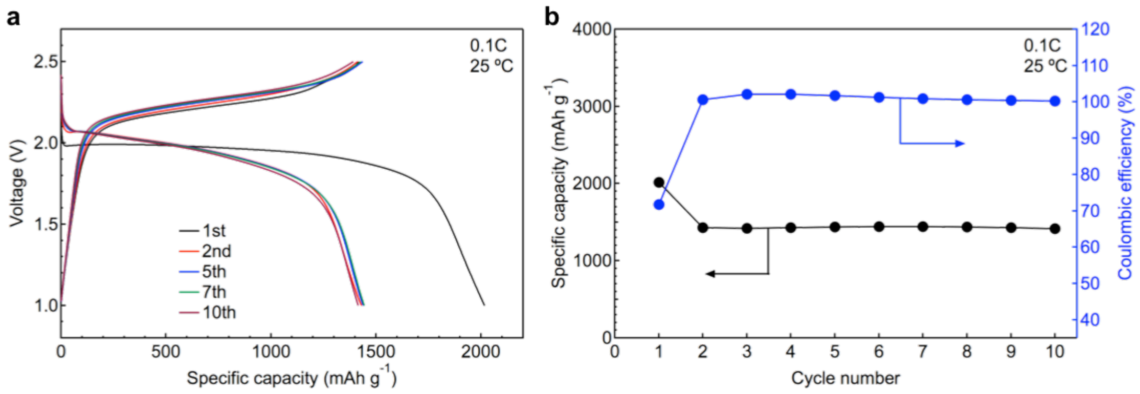

Supplementary Figure 16 | Discharge−charge performances for prolonged cycles. a, Discharge−charge profiles of a S/0.7Li(CB_9H_{10})−0.3Li($CB_{11}H_{12}$)/Li cell for a rate of 0.1C at 25 °C. **b,** Cycling performance for a rate of 0.1C at 25 °C.

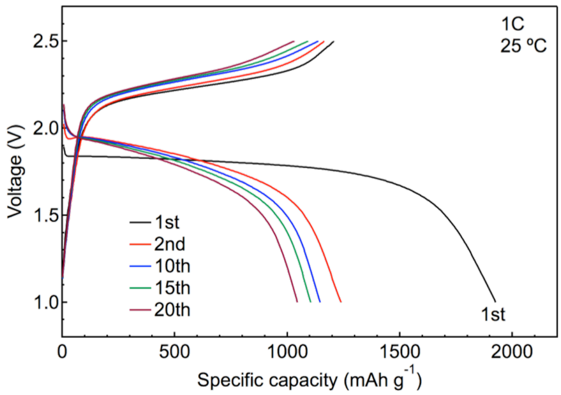

Supplementary Figure 17 | Discharge−charge profiles for prolonged cycles. Discharge−charge profiles of a S/0.7Li(CB_9H_{10})−0.3Li($CB_{11}H_{12}$)/Li cell for a rate of 1C at 25 °C.

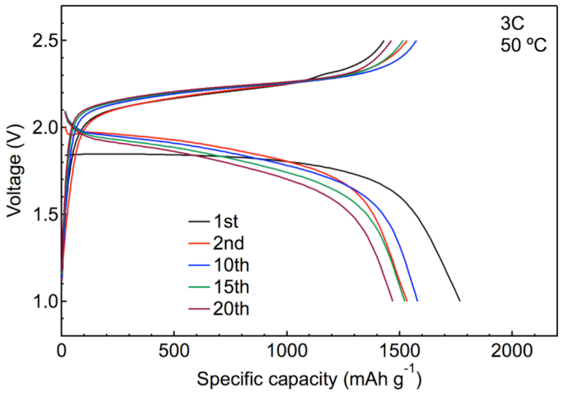

Supplementary Figure 18 | Discharge−charge profiles for prolonged cycles. Discharge−charge profiles of a S/0.7Li(CB_9H_{10})−0.3Li($CB_{11}H_{12}$)/Li cell for a discharging rate of 3C and a charging rate of 1C at 50 °C.

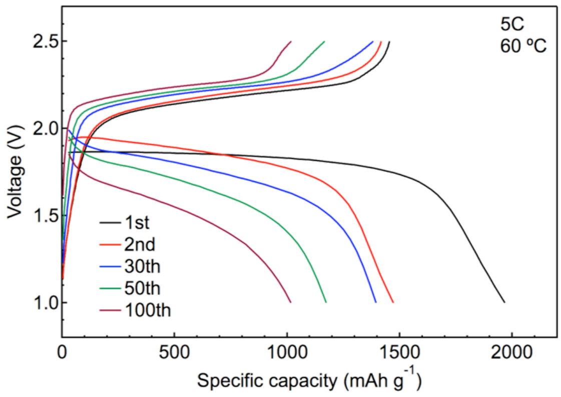

Supplementary Figure 19 | Discharge–charge profiles for prolonged cycles. Discharge-charge profiles of a S/0.7Li(CB_9H_{10})–0.3Li($CB_{11}H_{12}$)/Li cell for a discharging rate of 5C and a charging rate of 1C at 60°C.

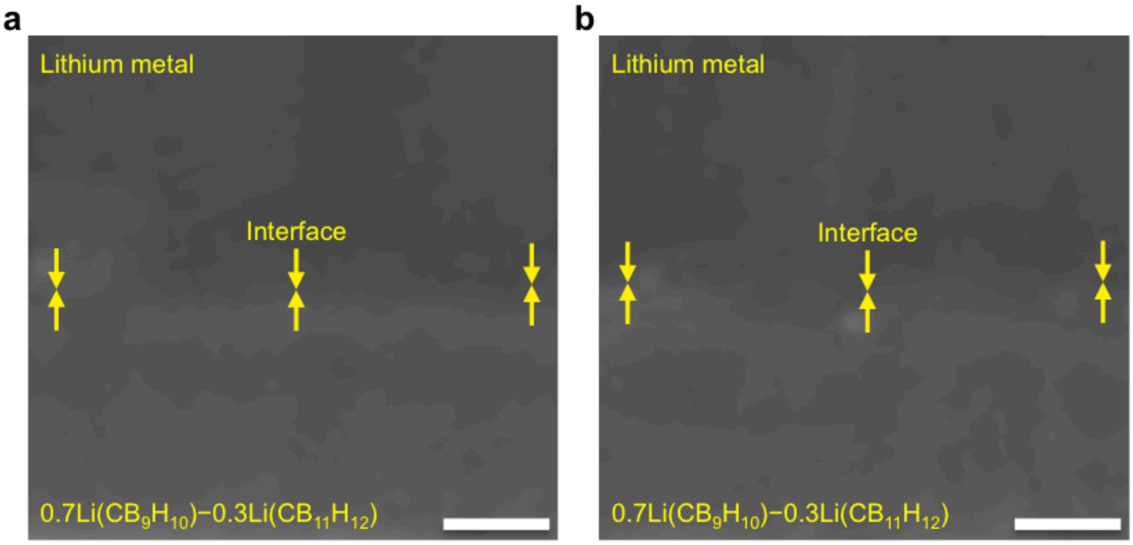

Supplementary Figure 20 | Stability of the 0.7Li(CB_9H_{10})−0.3Li($CB_{11}H_{12}$)/Li interface after cycling. a,b, FE−SEM images of the 0.7Li(CB_9H_{10})−0.3Li($CB_{11}H_{12}$)/Li interface for multiple measurement regions after 100 cycles. Scale bars, 20 μm.

Supplementary Table 1 | Phase transition temperatures (T_{trans}) of *closo*−type complex hydrides, lithium ion conductivities of low− and high−*T* phases, and activation energies of high−*T* phases. The phase transition temperature is defined as the temperature at which the phase transition finishes in the heating process or starts in the cooling process.

Material	T_{trans} (°C)	Phase	Ionic conductivity (S cm^{-1})	Activation Energy (kJ mol^{-1})	Reference
$Li(CB_9H_{10})$	90	Low−*T*	3.6×10^{-6} (25 °C)	−	1
		High−*T*	8.1×10^{-2} (110 °C)	28.9	1
$Li(CB_{11}H_{12})$	120	Low−*T*	5.2×10^{-5} (33 °C)	−	4
		High−*T*	2.0×10^{-1} (150 °C)	24.1	4
$Li_2(B_{12}H_{12})$	360	Low−*T*	2.5×10^{-8} (30 °C)	47.5	5
		−	−	−	−
$0.7Li(CB_9H_{10})$ $-0.3Li(CB_{11}H_{12})$	<r. t. [a]	High−*T*	6.7×10^{-3} (25 °C)	28.4	Present study
		High−*T*	8.5×10^{-2} (110 °C)		Present study

[a] r. t. means room temperature.

Supplementary Table 2 | Crystal systems, space groups, and lattice parameters of low− and high−*T* phases

Material	Phase	Crystal system	Space group	Lattice parameter (Å)	Reference
$Li(CB_9H_{10})$	Low−*T*	Hexagonal (r. t.)	$P3c1$ ($Z = 6$)	$a = 11.81(2)$ $c = 10.18(3)$	Present study
	High−*T*	Hexagonal (150 °C)	$P31c$ ($Z = 2$)	$a = 6.829$ $c = 10.754$	1
$Li(CB_{11}H_{12})$	Low−*T*	Orthorhombic (r. t.)	$Pca2_1$ ($Z = 4$)	$a = 9.667$ $b = 9.489$ $c = 9.727$	4
	High−*T*	Cubic (157 °C)	−	$a = 9.936$	4
$Li_2(B_{12}H_{12})$	Low−*T*	Cubic (r. t.)	$Pa{-}3$ ($Z = 4$)	$a = 9.577$	6
	High−*T*	Cubic (360 °C)	$Pa{-}3$ ($Z = 4$)	$a = 10.017$	7
$0.7Li(CB_9H_{10})$ $-0.3Li(CB_{11}H_{12})$	High−*T*	Hexagonal (r. t.)	$P31c$ ($Z = 2$)	$a = 6.86(1)$ $c = 10.77(1)$	Present study

Supplementary Table 3 | Quantitative values of circuit components from EIS analyses shown in Supplementary Fig. 13. The 0.7Li(CB_9H_{10})−0.3Li($CB_{11}H_{12}$)/Li interfacial resistance (Ω cm^2) was calculated by dividing R_2 by 2, and then normalizing the result by the surface area.

R_1 (Ω)	R_2 (Ω)	C_2 (F)
26.5	1.98	1.84×10^{-6}

Supplementary Table 4 | Interfacial resistances between solid electrolytes and lithium metal anodes measured using EIS analyses.

Solid electrolyte	Buffer layer	Interfacial resistance (Ω cm^2)	Reference
$0.7Li(CB_9H_{10})$−$0.3Li(CB_{11}H_{12})$	−	0.78	Present study
Al−substituted $Li_7La_3Zr_2O_{12}$	−	109	8
$Li_7La_3Zr_2O_{12}$	−	512	9
$Li_7La_{2.75}Ca_{0.25}Zr_{1.75}Nb_{0.25}O_{12}$	Al_2O_3	34	10 [a]
Al−contaminated $Li_{6.6}La_3Zr_{1.6}Ta_{0.4}O_{12}$	Au	380	11
$Li_{10}GeP_2S_{12}$	−	51 Ω [b]	12
$Li_{6.75}La_3Zr_{1.75}Ta_{0.25}O_{12}$	−	650 Ω [b]	13

[a] For ref. 10, the interfacial resistance was also evaluated using the DC method. Our experiments suggest that, for battery systems in which resistances of the solid electrolyte and solid electrolyte/electrode interface are extremely small, as in the present work, the evaluation of the interfacial resistance using the DC method should be reconsidered because low−external−resistance components, which are attributed to contributions of electrodes and cells, significantly affect the total resistance.

[b] The surface area was not reported.

Supplementary Note 1 | Structures of *closo*−type complex anions.

Closo-type materials contain so-called *closo*-type complex anions (such as $(B_{12}H_{12})^{2-}$, $(CB_{11}H_{12})^{-}$, and $(CB_9H_{10})^{-}$), in which the boron, carbon, and hydrogen atoms form unique covalent bonding patterns (boron−boron, boron−carbon, boron−hydrogen, and carbon−hydrogen), giving rise to a robust and stable cage−like polyanionic structure[14]. The $(CB_9H_{10})^{-}$ complex anions have a bicapped−square−antiprismatic structure (Supplementary Fig. 1a). The $(CB_{11}H_{12})^{-}$ complex anions have an icosahedral structure (Supplementary Fig. 1b).

Supplementary Note 2 | Structures and ionic conductivities of xLi(CB_9H_{10})−(1−x)Li($CB_{11}H_{12}$).

As the temperature increases from 25 to 90 °C, Li(CB_9H_{10}) (x = 0) displays a drastic jump in ionic conductivity, which originates from a transition to the high−T phase (Supplementary Fig. 5b). The Arrhenius plot of the ionic conductivities of 0.9Li(CB_9H_{10})−0.1Li($CB_{11}H_{12}$) (x = 0.1) exhibits no linear increase from 25 to 60 °C, indicating the incomplete stabilization of the high−T phase. In addition, 0.5Li(CB_9H_{10})−0.5Li($CB_{11}H_{12}$) (x = 0.5) contains a massive amount of other impurity phases[15], which is presumably due to the high $(CB_{11}H_{12})^-$ content exceeding the solid−solution region (Supplementary Fig. 5a).

Supplementary Note 3 | Structure change of starting materials after mechanical ball−milling.

Neither starting material displays structural changes after mechanical ball−milling (Supplementary Fig. 7). The broadened XRD peaks after mechanical ball−milling are presumably due to decreased crystallinity.

Supplementary Note 4 | Effects of ball−milling and heat−treatment on conductivity.

The impedance results of ball−milled $Li(CB_9H_{10})$ and heat−treated $0.7Li(CB_9H_{10})$−$0.3Li(CB_{11}H_{12})$ indicate that the size has a negligible effect on ionic conductivity (Supplementary Fig. 10).

Supplementary Note 5 | *Closo*−type complex hydride ionic conductors.

Various *closo*−type complex hydrides have been investigated for sodium ion conductors[16-18]. In general, sodium−based complex hydrides have crystal structures, solid−solution regions, and properties different from those of their lithium−based counterparts[15,19,20]. Moreover, differences between the properties of lithium− and sodium−based compounds have been confirmed for most other battery materials. These differences are attributed to differences in the properties, such as size and polarizability[21], of the constituent cations. Therefore, different design principles based on material properties are needed for lithium− and sodium−based complex hydride ionic conductors.

Supplementary Note 6 | Lithium diffusion coefficient of 0.7Li(CB_9H_{10})–0.3Li($CB_{11}H_{12}$).

The activation energy of the self–diffusion coefficient was 30.2 kJ mol^{-1} (Fig. 2c and Supplementary Fig. 11), which is comparable to that of ionic conductivity. The conductivity diffusion coefficient (D_σ) shown in Fig. 2c was calculated using the Nernst–Einstein equation, shown in equation (1):

$$D_\sigma = \frac{k_B T}{n(ze)^2}\sigma \tag{1}$$

where D_σ is the conductivity diffusion coefficient, k_B is the Boltzmann constant, T is the absolute temperature, n is the concentration of mobile ions, z is the valence of the ions, e is the elemental charge, and σ is the conductivity. The lithium ion concentration (n = 4.6 × 10^{27} m^{-3}) was calculated from the unit cell volume (V/Z = 219 Å^3) and the composition of 0.7Li(CB_9H_{10})–0.3Li($CB_{11}H_{12}$).

Supplementary Note 7 | EIS analyses of a Li/0.7Li(CB_9H_{10})−0.3Li($CB_{11}H_{12}$)/Li cell

The ionic conductivity (6.7×10^{-3} S cm^{-1}) calculated from the high−frequency x−intercept (R_1 in Supplementary Fig. 13 and Supplementary Table 3) of the semicircle agrees well with that (6.7×10^{-3} S cm^{-1}) estimated from the Au/0.7Li(CB_9H_{10})−0.3Li($CB_{11}H_{12}$)/Au cell (Fig. 2a). Empirically, capacitances of the bulk, grain boundary, and interface regions for ionic conduction are on the order of 10^{-12}, 10^{-9}, and 10^{-6} F, respectively[22]. According to our fitting, the capacitance component (C_2) of the semicircle is on the order of 10^{-6} F (Supplementary Table 3), and hence the observed impedance (R_2) corresponds to an electrode/electrolyte interfacial resistance.

Supplementary Note 8 | Discharge−charge performances for prolonged cycles

The single plateau in the discharge process indicates that the formation of soluble long−chain polysulfides, which is a typical problem for liquid−electrolyte−based batteries[23], is avoided (Supplementary Fig. 16a). In this study, the cathode composites were prepared by simple hand−mixing of a S−C composite and the complex hydride solid electrolyte. The cell performances can be further enhanced by improving the fabrication technology used for the S−C−complex hydride composite[24].

Supplementary References

1. Tang, W. S. *et al.* Liquid-like ionic conduction in solid lithium and sodium monocarba-*closo*-decaborates near or at room temperature. *Adv. Energy Mater.* **6,** 1502237 (2016).

2. Stejskal, E. O. & Tanner J. E. Spin diffusion measurements: Spin echoes in the presence of a time-dependent field gradient. *J. Chem. Phys.* **42,** 288−292 (1965).

3. Hayamizu, K. & Aihara Y. Lithium ion diffusion in solid electrolyte $(Li_2S)_7(P_2S_5)_3$ measured by pulsed-gradient spin-echo ^{7}Li NMR spectroscopy. *Solid State Ionics* **238,** 7−14 (2013).

4. Tang, W. S. *et al.* Unparalleled lithium and sodium superionic conduction in solid electrolytes with large monovalent cage-like anions. *Energy Environ. Sci.* **8,** 3637−3645 (2015).

5. Kim, S. *et al.* Fast lithium-ion conduction in atom-deficient *closo*-type complex hydride solid electrolytes. *Chem. Mater.* **30,** 386−391 (2018).

6. Her, J. *et al.* Crystal structure of $Li_2B_{12}H_{12}$: a possible intermediate species in the decomposition of $LiBH_4$. *Inorg. Chem* **47,** 9757−9759 (2008).

7. Paskevicius, M. *et al.* First-order phase transition in the $Li_2B_{12}H_{12}$ system. *Phys. Chem. Chem. Phys.* **15,** 15825−15828 (2013).

8. Cheng, L. *et al.* The origin of high electrolyte-electrode interfacial resistances in lithium cells containing garnet type solid electrolytes. *Phys. Chem. Chem. Phys.* **16,** 18294−18300 (2014).

9. Sharafi, A. *et al.* Characterizing the Li–$Li_7La_3Zr_2O_{12}$ interface stability and kinetics as a function of temperature and current density. *J. Power Sources* **302,** 135−139 (2016).

10. Han, X. *et al.* Negating interfacial impedance in garnet-based solid-state Li metal batteries. *Nat. Mater.* **16,** 572−579 (2017).

11. Tsai, C. *et al.* $Li_7La_3Zr_2O_{12}$ interface modification for Li dendrite prevention. *ACS Appl. Mater. Interfaces* **8,** 10617−10626 (2016).

12. Oh, G. *et al.* Bulk-type all solid-state batteries with 5 V class $LiNi_{0.5}Mn_{1.5}O_4$ cathode and $Li_{10}GeP_2S_{12}$ solid electrolyte. *Chem. Mater.* **28,** 2634−2640 (2016).

13. Liu, T. *et al.* Achieving high capacity in bulk-type solid-state lithium ion battery based on $Li_{6.75}La_3Zr_{1.75}Ta_{0.25}O_{12}$ electrolyte: Interfacial resistance. *J. Power Sources* **324,** 349−357 (2016).

14. Hansen, B. R. S. *et al.* Metal boranes: progress and applications. *Coord. Chem. Rev.* **323,** 60−70 (2016).

15. Tang, W. S. *et al.* Stabilizing superionic-conducting structures via mixed-anion solid solutions of monocarba-*closo*-borate salts. *ACS Energy Lett.* **1,** 659−664 (2016).

16. Duchêne, L. *et al.* A stable 3 V all–solid–state sodium–ion battery based on a closo-borate electrolyte. *Energy Environ. Sci.* **10,** 2609−2615 (2017).

17. Duchêne, L. *et al.* A highly stable sodium solid-state electrolyte based on a dodeca/deca–borate equimolar mixture. *Chem. Commun.* **53,** 4195−4198 (2017).

18. Brighi, M. *et al.* A mixed anion hydroborate/carba–hydroborate as a room temperature Na–ion solid electrolyte. *J. Power Sources* **404,** 7−12 (2018).

19. Orimo, S. *et al.* Complex hydrides for hydrogen storage. *Chem. Rev.* **107,** 4111−4132 (2007).

20. Paskevicius, M. *et al.* Metal borohydrides and derivatives - synthesis, structure and properties. *Chem. Soc. Rev.* **46,** 1565−1634 (2017).

21. Yabuuchi, N., Kubota K., Dahbi M. & Komaba S. Research development on sodium-ion batteries. *Chem. Rev.* **114,** 11636−11682 (2014).

22. Felice, C. J. *et al.* Impedance microbiology: quantification of bacterial content in milk by means of capacitance growth curves. *J. Microbiol. Meth.* **35,** 37−42 (1999).

23. Bresser, D., Passerini S. & Scrosati B. Recent progress and remaining challenges in sulfur-based lithium secondary batteries – a review. *Chem. Commun.* **49,** 10545−10562 (2013).

24. Suzuki, K. *et al.* High cycle capability of all-solid-state lithium–sulfur batteries using composite electrodes by liquid-phase and mechanical mixing. *ACS Appl. Mater. Interfaces* **1,** 2373−2377 (2018).
